# Laparoscopic Peritoneal Flap Closure for Internal Hernia Associated with Colostomy: Two Case Reports

**DOI:** 10.70352/scrj.cr.25-0831

**Published:** 2026-06-09

**Authors:** Ryo Kanoda, Hisahito Endo, Masanori Katagata, Takuro Matsumoto, Daisuke Ujiie, Shun Chida, Hiroyuki Hanayama, Hirokazu Okayama, Zenichiro Saze, Tomoyuki Momma, Koji Kono

**Affiliations:** Department of Gastrointestinal Tract Surgery, Fukushima Medical University School of Medicine, Fukushima, Fukushima, Japan

**Keywords:** internal hernia associated with colostomy, peritoneum, laparoscopic peritoneal flap closure

## Abstract

**INTRODUCTION:**

Internal hernia associated with colostomy (IHAC) is a rare but serious complication after colorectal surgery, especially when performed via the intraperitoneal route. Optimal treatment strategies remain unclear.

**CASE PRESENTATION:**

We report 2 cases of recurrent bowel obstruction caused by IHAC following laparoscopic Hartmann’s procedure or laparoscopic abdominoperineal resection (APR) performed via the intraperitoneal route in patients aged 74 and 83 years. Both patients underwent laparoscopic peritoneal flap closure (LPFC), in which the peritoneum was dissected and sutured to the lifted colon to close the lateral space of the colostomy. The procedures were successful, with favorable postoperative courses and no recurrence of IHAC in either case.

**CONCLUSIONS:**

The present cases demonstrate that LPFC is a safe and effective technique for repairing IHAC, particularly when conventional methods, such as omental patching or extraperitoneal stoma creation, are not feasible. This approach may also serve as a preventive measure against IHAC if performed during the primary surgery.

## Abbreviations

APR
: abdominoperineal resection
ICG
: indocyanine green
IHAC
: internal hernia associated with colostomy
LPFC
: laparoscopic peritoneal flap closure
TAPP
: transabdominal preperitoneal repair
USAD
: ultrasonically activated device

## INTRODUCTION

There are 2 main approaches for colostomy: the extraperitoneal route and the intraperitoneal route, in which the colon is brought directly to the abdominal wall through the abdominal cavity. The intraperitoneal route is technically simpler and is therefore widely adopted in laparoscopic colorectal surgery. However, it carries a risk of internal herniation through the lateral peritoneal defect (hernia orifice) adjacent to the stoma, referred to as an IHAC, which may lead to bowel obstruction^1)^ (**Fig. 1**).

**Fig. 1:**
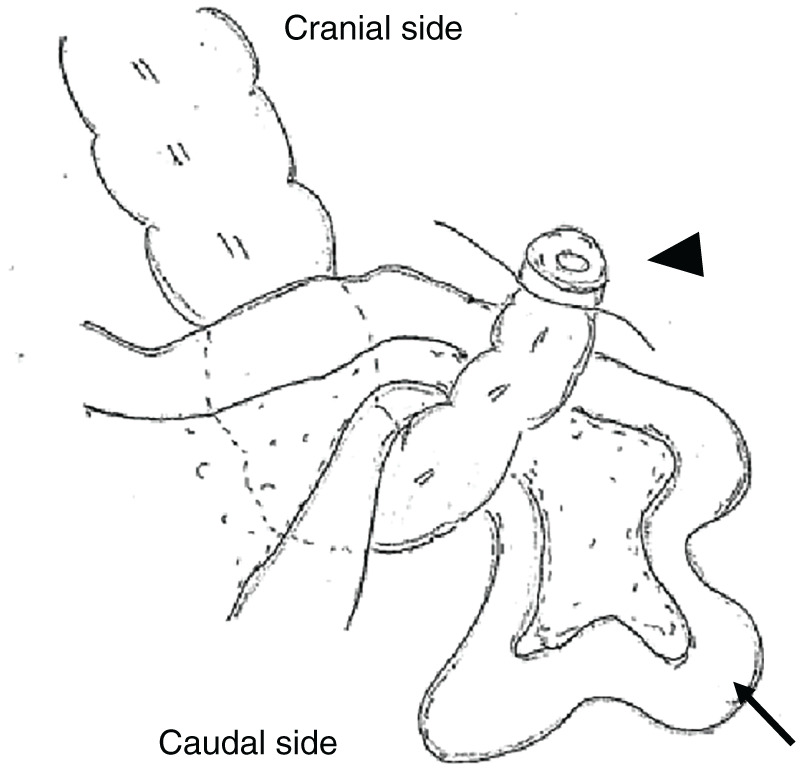
Schematic illustration of IHAC. The arrowhead indicates the sigmoid colostomy, and the arrow indicates the small intestine herniating into the lateral space. IHAC, internal hernia associated with colostomy

Herein, we report 2 cases of recurrent bowel obstruction caused by IHAC following laparoscopic APR or Hartmann’s procedure performed via the intraperitoneal route. Both cases were successfully managed using LPFC of the hernia orifice, resulting in favorable postoperative outcomes.

## CASE PRESENTATION

### Case 1

A 74-year-old man (165 cm, 62 kg) with a history of left thalamic hemorrhage underwent laparoscopic APR with lateral lymph node dissection for rectal cancer. A sigmoid colostomy was performed via the intraperitoneal route. Soon after surgery, the patient experienced recurrent episodes of small bowel obstruction, which temporarily improved with fasting. Contrast-enhanced CT revealed herniation of the small intestine into the lateral space between the lifted sigmoid colon and the abdominal wall, causing intermittent obstruction (**Fig. 2**). Following 5 recurrent episodes of intestinal obstruction, LPFC of the hernia orifice was performed 12 months after the initial surgery.

**Fig. 2:**
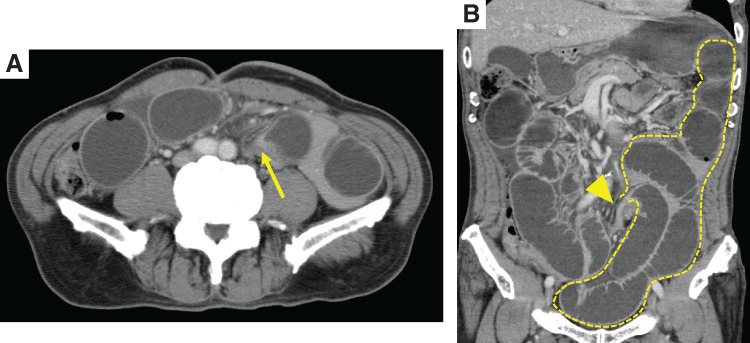
(**A**, **B**) Dilatation of the small intestine is observed. A stricture point (arrow) is noted near the colostomy site (arrowhead). The dotted line represents the small intestine located lateral to the colostomy, forming an internal hernia.

During the surgery, 12-mm trocars were placed at the umbilicus and the right lower abdomen, and a 5-mm trocar was placed at the right lateral abdomen. An additional 5-mm trocar was inserted slightly to the left of the suprapubic area to facilitate suturing. The small intestine was adhered to the pelvic floor but not to the lifted colon or the dissected retroperitoneum, which exhibited regenerated peritoneum. It extended laterally around the lifted colon, which was considered the cause of the bowel obstruction (**Fig. 3A**). The peritoneum cranial to the common iliac artery was dissected using an USAD while preserving the subperitoneal tissue. The dissected peritoneum was expanded to an adequate size and used to cover the hernia orifice of the stoma (**Fig. 3B** and **3C**). It was then sutured continuously to the lifted colon using 3-0 barbed nonabsorbable sutures (V-Loc; Medtronic, Minneapolis, USA) (**Fig. 3D**). No leakage was observed upon water infusion, and the durability of the peritoneum was evaluated by repositioning the patient to position the intestinal tract cranially in relation to the peritoneum. An adhesion barrier (AdSpray; TERUMO JAPAN, Tokyo, Japan) was applied to the dissected area (**Fig. 3E**), and the surgery was completed. The operative time was 1 h and 45 min, with no blood loss. Abdominal discomfort resolved the following day, and the patient was discharged without complications. There was no recurrence of bowel obstruction during the 18 months following LPFC, until the patient died from recurrent rectal cancer.

**Fig. 3:**
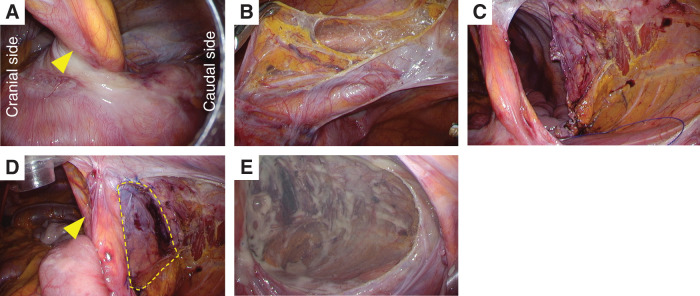
(**A**) Internal hernia orifice around the colostomy (arrowhead). (**B**) Peritoneal dissection is performed to create a flap. (**C**) The surgical field after completion of peritoneal dissection. (**D**) The peritoneal flap (dotted line) was sutured to the lifted colon (arrowhead). (**E**) An adhesion barrier agent was applied to the dissected peritoneal area.

### Case 2

An 83-year-old man (164 cm, 55 kg) with a history of chronic kidney disease on hemodialysis and paroxysmal atrial fibrillation underwent a laparoscopic Hartmann’s procedure via the intraperitoneal route and a right hemicolectomy for multiple colon cancers in the sigmoid, transverse, and cecal regions. Six months after surgery, he presented with abdominal pain and vomiting, and was diagnosed with small bowel obstruction caused by the small intestine herniating through the orifice between the lifted sigmoid colon and the abdominal wall (**Fig. 4**). The symptoms spontaneously resolved within 1 day; however, similar episodes recurred 3 times within the same month, prompting surgical intervention.

**Fig. 4:**
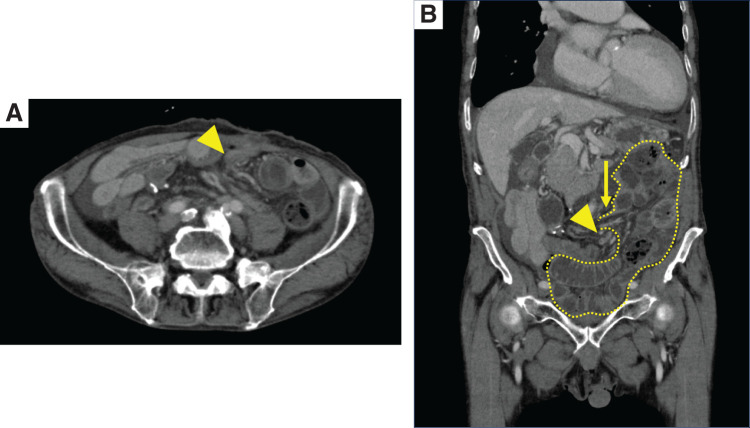
(**A**, **B**) Dilatation of the small intestine is observed. A caliber change (arrow) is noted near the colostomy site (arrowhead). The dotted line represents the small intestine located lateral to the colostomy, forming an internal hernia.

Laparoscopic surgery was performed using 4 ports: 12-mm trocars were placed at the umbilicus and above the pubis, a 5-mm trocar was placed in the right lateral abdomen, and an additional 5-mm trocar was placed between the 12- and 5-mm ports. On observation of the abdominal cavity, the small intestine was seen passing laterally around the lifted colon (**Fig. 5A**). The regenerated peritoneum at the retroperitoneal dissection site showed no adhesions. The peritoneum over the hernia orifice was dissected using an USAD and mobilized to cover the defect (**Fig. 5B** and **5C**). The blood flow of the dissected peritoneum was confirmed using ICG fluorescence imaging (**Fig. 5D** and **5E**). The mobilized peritoneum was sutured continuously to the abdominal wall and the lifted colon using 3-0 barbed nonabsorbable sutures, effectively closing the hernia orifice. The operative time was 1 h and 56 min, with no blood loss.

**Fig. 5:**
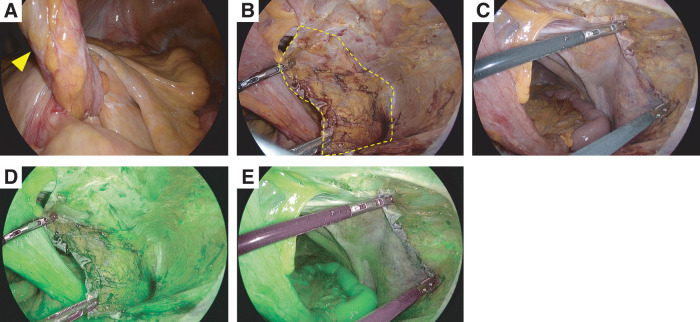
(**A**) An internal hernia is observed around the colostomy site (arrowhead). (**B**, **C**) Peritoneal dissection was performed to create a flap (dotted line). (**D**, **E**) ICG imaging confirmed adequate blood flow in the peritoneum. ICG, indocyanine green

Postoperatively, the patient’s reported abdominal discomfort resolved on the first day, and there was no recurrence of bowel obstruction during 18 months of follow-up. Postoperative CT at 6 months after LPFC showed no herniation of the small intestine from the cranial side of the colostomy (**Fig. 6**).

**Fig. 6:**
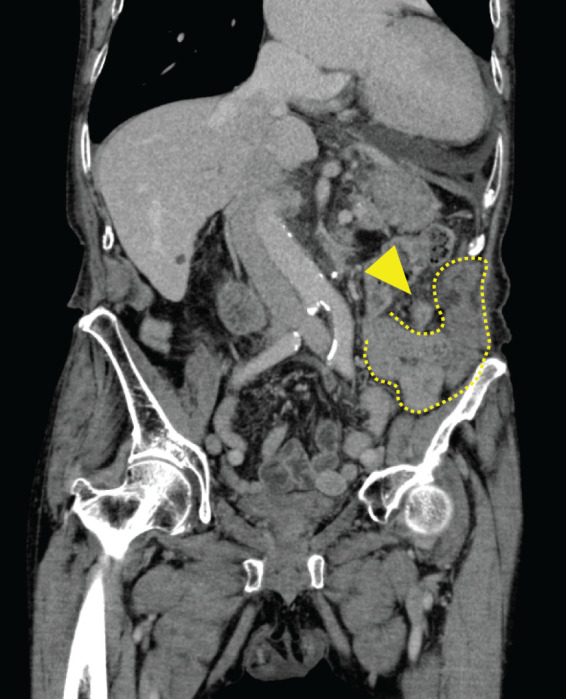
Postoperative CT after LPFC showed no herniation of the small intestine from the cranial side of the colostomy (arrowhead), while the small intestine was observed lateral to the colostomy from the caudal side (dotted line). LPFC, laparoscopic peritoneal flap closure

## DISCUSSION

To the best of our knowledge, this is the first report on peritoneal flap closure for the repair of IHAC. The increasing adoption of laparoscopic colorectal surgery has brought technical advantages but has also produced unique postoperative complications, such as IHAC, underscoring the need for innovative and safe repair methods.

Stoma creation routes have been conventionally divided into intraperitoneal and extraperitoneal approaches.^2)^ The intraperitoneal route is often preferred in laparoscopic procedures due to its technical simplicity and shorter operative time. However, it may predispose patients to internal hernias due to fewer postoperative adhesions than open surgery. Although the reduction in adhesions is beneficial, it paradoxically increases the mobility of the small bowel, thereby increasing the risk of herniation through defects around the stoma site. Previous reports have indicated that the incidence of IHAC is approximately 1.5%.^3)^ Däster et al.^4)^ reported that the incidence of asymptomatic internal hernias after colorectal cancer surgery is approximately 27%, suggesting that asymptomatic IHAC may be relatively common.

Current treatment strategies for IHAC include stoma reconstruction via the extraperitoneal route,^3)^ fixation of the lifted colon to the abdominal wall,^5)^ or filling the lateral hernia space with omentum^3)^. However, these approaches may not be feasible in all patients. A shorter lifted colon can preclude extraperitoneal stoma creation or secure fixation, whereas insufficient omentum limits the use of omental packing. In addition, reports of internal hernias following extraperitoneal stoma formation^6,7)^ suggest that this approach cannot entirely prevent their occurrence. Our approach using LPFC addresses these limitations by leveraging the inherent properties of the peritoneum. A PubMed search using the keywords “internal hernia” and “colostomy” yielded no reports describing a similar approach.

From a mechanistic perspective, IHAC is primarily caused by increased small bowel mobility in combination with the presence of a lateral potential space around the lifted colon, rather than by a structural weakness of the abdominal wall. Preventive strategies such as the extraperitoneal route aim to avoid the creation of this potential space; however, reports of IHAC even after extraperitoneal stoma creation suggest that this approach does not completely eliminate the risk when a residual or newly formed space persists. In contrast, LPFC is conceptually a space-eliminating technique that obliterates the hernia orifice after it has formed, rather than reinforcing the abdominal wall. Thus, LPFC and extraperitoneal stoma creation can be regarded as complementary approaches targeting the same pathophysiological mechanism at different stages. IHAC can therefore be more appropriately understood as a “space-driven” rather than a “pressure-driven” pathology. Within this conceptual framework, LPFC functions as a space-eliminating technique that addresses the underlying mechanism by obliterating the potential space that permits bowel herniation.

LPFC is usually a surgical technique that uses the peritoneal dissection method applied in TAPP. Compared with TAPP, it involves a more extensive peritoneal dissection to create a peritoneal flap for hernia orifice closure. The use of peritoneal flaps has also been reported as a method for preventing perineal hernia, in which a peritoneal flap derived from the bladder is created after APR to close the pelvic inlet.^8,9)^

The peritoneum, with its rich vascularity and marked regenerative capacity, serves as an ideal autologous tissue for hernia repair. It possesses an abundant vascular network,^10)^ ensuring adequate blood flow even after dissection and thereby minimizing the risk of tissue necrosis. A review of the literature revealed no reported cases of postoperative peritoneal necrosis following TAPP, further suggesting that the peritoneum benefits from a robust blood supply. In our study, adequate peritoneal blood flow was confirmed in Case 2 by ICG fluorescence imaging. Furthermore, the peritoneum exhibits a high regenerative capacity. Rapid migration and proliferation of mesothelial cells typically lead to early re-epithelialization within a few days, promoting effective healing, with initial peritoneal regeneration generally completed within 7 to 8 days.^11)^ Accordingly, even if the peritoneum was dissected during the initial colorectal cancer surgery, it is likely to have regenerated by the time of reoperation for IHAC.

The gap between the lifted colon and the sutured peritoneum may indicate a risk of internal hernia. To address this, we ascertained the functionality of the peritoneal flap by injecting saline from its cranial side after suturing and confirming the absence of leakage. Furthermore, during flap creation, we extensively dissected the outer peritoneum cranially, creating a downward slope to prevent the small intestine from resting on the flap and allowing it to fall into the pelvic cavity. This technique minimizes potential hernia orifices and spaces for small bowel herniation, thereby reducing the risk of hernia formation.

The advantages of LPFC include avoiding foreign materials such as mesh, which reduces the risks of infection and foreign body reaction. This approach is particularly beneficial in contaminated or potentially infected fields, such as those associated with stoma sites. Moreover, LPFC is adaptable to numerous clinical scenarios, including a shorter lifted colon, insufficient omentum, and large hernia defects, making it a versatile option.

Nonetheless, LPFC has limitations. Severe adhesions between the peritoneum and bowel may complicate safe dissection, increasing operative time and the risk of hemorrhage or organ injury. Special attention must be paid to preventing injury to the ureter and gonadal vessels. Future studies should evaluate the reproducibility and safety of LPFC in larger patient cohorts and in diverse surgical settings.

With regard to mechanical strength and long-term durability, LPFC differs conceptually from mesh-based hernia repair techniques such as TAPP. In inguinal hernia repair, mesh reinforcement is essential to withstand the continuous intra-abdominal pressure applied to the abdominal wall defect. In contrast, IHAC is not primarily a pressure-driven hernia but results from increased mobility of the small bowel and the presence of a lateral internal space around the lifted colon. Therefore, the objective of LPFC is not to reinforce the abdominal wall but to eliminate the orifice to the potential space that allows bowel herniation. Similarly, although peritoneal flaps combined with adjacent organs such as the bladder have been used to achieve secure closure of large pelvic defects after APR, the defect associated with IHAC is relatively limited, and closure using a peritoneal flap alone may be sufficient to prevent bowel descent into the pelvic cavity. Nevertheless, the long-term durability of an autologous peritoneal flap remains uncertain. Compared with mesh repair, regenerated peritoneum may theoretically undergo stretching over time, potentially leading to late recurrence. Because the present report includes only 2 cases with a limited follow-up period, the durability of LPFC could not be fully evaluated. Although no recurrence was observed during follow-up, these findings should be interpreted with caution, and long-term follow-up studies with larger cohorts are required to clarify the true durability and recurrence risk of this technique.

In our 2 cases, no recurrence of internal hernia or bowel obstruction was observed during follow-up, suggesting durable repair efficacy. Although adhesion barriers were applied, the role of postoperative adhesions in preventing hernia recurrence remains unclear and warrants further investigation.

As minimally invasive approaches such as laparoscopic and robotic surgeries become standard, the incidence of postoperative adhesions decreases, which may paradoxically increase the risk of internal hernias, including IHAC. At the same time, the technical complexity of extraperitoneal stoma creation increases with minimally invasive surgeries, underscoring the need for alternative prophylactic and therapeutic strategies. LPFC is a promising method that utilizes the biological properties of the peritoneum for safe and effective autologous tissue repair. Further studies are required to validate its long-term outcomes and to clarify its role relative to other techniques.

## CONCLUSIONS

We reported 2 cases of IHAC successfully treated using a peritoneal flap. Both patients demonstrated sustained symptomatic improvement without recurrence, suggesting LPFC to be a feasible and safe option for IHAC repair, even in cases where conventional methods are not applicable.
